# Can Biomarkers Predict Kidney Function Recovery and Mortality in Patients with Critical COVID-19 and Acute Kidney Injury?

**DOI:** 10.3390/diagnostics15151960

**Published:** 2025-08-05

**Authors:** Noemí Del Toro-Cisneros, José C. Páez-Franco, Miguel A. Martínez-Rojas, Isaac González-Soria, Juan Antonio Ortega-Trejo, Hilda Sánchez-Vidal, Norma A. Bobadilla, Alfredo Ulloa-Aguirre, Olynka Vega-Vega

**Affiliations:** 1 Department of Nephrology and Mineral Metabolism, Instituto Nacional de Ciencias Médicas y Nutrición Salvador Zubirán, Mexico City 14080, Mexico; noemi_3090@hotmail.com (N.D.T.-C.); mikemarm_93@live.com.mx (M.A.M.-R.); igsoria23@gmail.com (I.G.-S.); jant.ortega@hotmail.com (J.A.O.-T.); nab@iibiomedicas.unam.mx (N.A.B.); 2 Red de Apoyo a la Investigación, Universidad Nacional Autónoma de México, Instituto Nacional de Ciencias Médicas y Nutrición Salvador Zubirán, Mexico City 14080, Mexico; paez@cic.unam.mx (J.C.P.-F.); hilda.sanchezv@incmnsz.mx (H.S.-V.); aulloaa@unam.mx (A.U.-A.); 3 Molecular Physiology Unit, Instituto de Investigaciones Biomédicas, Universidad Nacional Autónoma de México, Mexico City 14080, Mexico

**Keywords:** COVID-19, acute kidney injury, biomarkers, mortality, renal recovery

## Abstract

**Background/Objectives**: COVID-19 is a systemic viral infection that may lead to serious complications including acute kidney injury that requires kidney replacement therapy. The primary aim of this study was to evaluate urinary SerpinA3 (uSerpinA3) excretion as a biomarker of kidney recovery at 90 days, and the mortality in patients with critical COVID-19 and AKI requiring kidney replacement therapy (KRT). **Methods**: The study included patients with critical COVID-19 on invasive mechanical ventilation (IMV) requiring KRT. Blood and urine samples were obtained when KRT was initiated (day zero), and thereafter on days 1, 3, 7, and 14 post-replacement. uSerpinA3, kidney injury molecule-1 (uKIM-1), and neutrophil gelatinase-associated lipocalin (uNGAL) were measured in urine, and interleukin-6 (IL-6), interleukin-10 (IL-10), and tumor necrosis factor alpha (TNF-α) in peripheral blood. In addition, metabolomics in sample days zero and 3, and in the survivors on sample day 90 was performed by employing gas chromatography coupled with mass spectrometry. **Results**: A total of 60 patients were recruited, of whom 29 (48%) survived hospitalization and recovered kidney function by day 90. In the survivors, 79% presented complete recovery (CRR) and the remaining (21%) recovered partially (PRR). In terms of uSerpinA3, levels on days 7 and 14 predicted CRR, with AUC values of 0.68 (*p* = 0.041) and 0.71 (*p* = 0.030), respectively, as well as mortality, with AUC values of 0.75 (*p* = 0.007) and 0.76 (*p* = 0.015), respectively. Among the other biomarkers, the excretion of uKIM-1 on day zero of KRT had a superior performance as a CRR predictor [(AUC, 0.71 (*p* = 0.017)], and as a mortality predictor [AUC, 0.68 (*p* = 0.028)]. In the metabolomics analysis, we identified four distinct profiles; the metabolite that maintained statistical significance in predicting mortality was p-cresol glucuronide. **Conclusions**: This study strongly suggests that uSerpinA3 and uKIM-1 can predict CRR and mortality in patients with critical COVID-19 and AKI requiring KRT. Metabolic analysis appears promising for identifying affected pathways and their clinical impact in this population.

## 1. Introduction

The prevalence of acute kidney injury (AKI) in patients hospitalized due to COVID-19 is extremely variable, with our center reporting a prevalence of 30% [1]. However, in critically ill patients hospitalized in the intensive care unit (ICU), AKI is more frequent, developing in up to 90% of cases, particularly in patients requiring invasive mechanical ventilation (IMV) [2,3]. Among COVID-19 patients on IMV that develop AKI, the rate of kidney replacement therapy (KRT) requirement has been shown to reach ~35% [3,4]. The rates of kidney function recovery in these AKI patients range between 67% and 80% [5,6]. In the medium term, after recovering renal function, these patients develop an accelerated decrease in glomerular filtration rate (GFR) that can reach 11 mL/min/1.73 m^2^, in comparison with AKI patients without associated COVID-19 [7].

Measurement of serum creatinine (sCr) has serious limitations, particularly when quantifying renal recovery; in fact, several studies have demonstrated the association between AKI and chronic kidney disease (CKD) progression, even in cases with previously known baseline sCr levels [8]. These findings prompted the need to search for other diagnostic methods to detect renal abnormalities after an AKI episode. Thus, in the last few decades, several biomarkers have been studied to predict AKI, KRT requirements, renal function recovery, and mortality [9,10,11]. Nevertheless, in the context of COVID-19, the latter two outcomes have been incompletely evaluated.

SerpinA3 is a serine protease inhibitor that is expressed in response to inflammatory cytokines and that regulates the activity of different proteases such as neutrophil-derived cathepsin G [12]. In addition to its role in inflammation, SerpinA3 has also been shown to participate in the regulation of angiogenesis, apoptosis, oxidative stress, cell proliferation, and fibrosis in mouse models of corneal burns, although all the above processes are also related to the transition from AKI to CKD [13]. We recently evaluated urinary SerpinA3 (uSerpinA3) in an experimental transition model from AKI to CKD, in which a temporal progressive increase that preceded the elevation of classic CKD markers, such as proteinuria and fibrosis, was observed, with the greater the excretion of uSerpinA3, the greater fibrosis. In addition, the abnormal presence of uSerpinA3 was detected in patients with different kidney diseases, and again, a significant correlation between the uSerpinA3 and the degree of fibrosis was observed [13]. We recently studied the temporal evolution of uSerpinA3 during the first year after the start of immunosuppressive treatment in 60 patients with lupus nephropathy who presented an acute flare of the disease and whether there was a relationship with the degree of therapeutic response at 12 months. We found that uSerpinA3 can identify patients who had a favorable response to treatment, as they showed an early reduction in uSerpinA3, while non-responders maintained high levels of uSerpinA3 excretion throughout the follow-up [14].

Based on our previous findings [13,14] the primary aim of this study was to evaluate uSerpinA3 as a biomarker of mortality and renal recovery at 90 days of KRT in patients with critical COVID-19 and AKI warranting KRT. Our secondary aims were to evaluate the performance of other serum biomarkers, including interleukin-6 (IL-6), interleukin-10 (IL-10) and tumor necrosis factor-α (TNF-α), and urinary neutrophil-associated gelatinase lipocalin (uNGAL) and kidney injury molecule-1 biomarkers, as well as some metabolic changes, as predictors of the same outcomes.

## 2. Materials and Methods

The study was conducted at a tertiary care level health institution in Mexico City (Instituto Nacional de Ciencias Médicas y Nutrición Salvador Zubirán). Written informed consent was obtained from each patient included in the study. The study protocol conforms to the ethical guidelines of the 1975 Declaration of Helsinki, and all experimental protocols were approved by the Ethics Committee (NMM-3325-20-20-1) and the Bioethics Committee (CEI-011-20160627) of the institution. The cohort included all adult patients with a positive polymerase chain reaction (PCR) test for SARS-CoV-2 admitted between March/2020 and February/2022 to the intensive care unit (ICU), who presented with IMV and AKI requiring KRT. Patients with advanced chronic kidney disease (estimated glomerular filtration rate < 30 mL/min/1.73 m^2^) and those with a prior kidney transplant were excluded. Control patients with COVID-19 on IMV, but with no AKI were also recruited and paired by age, sex, and comorbidities with the surviving patients on KRT.

### 2.1. Data Collection and Laboratory Measurements

The collected variables included demographic data, previous comorbidities, laboratory parameters at KRT initiation, the use of vasoactive drugs (pressors or inotropics), drugs administered in the ICU, and the patients’ course. Urinary biomarkers (uNGAL, uKIM-1, uSerpinA3) were measured on day zero (the day KRT was initiated) and subsequently on days 1, 3, 7, and 14 after the beginning of KRT. In those patients with AKI and KRT who survived, uNGAL, uKIM-1, uSerpinA3 were also measured 90 days post-KRT. Serum biomarkers, IL-6, IL-10 and TNF-α, were only measured on day zero. Urinary concentrations of NGAL and KIM-1 were analyzed with commercially available ELISA kits (DY1757-05 for NGAL, and DY1750B for KIM-1; R&D Systems, Minneapolis, MN, USA), following the manufacturer’s instructions (R&D Systems). SerpinA3 was analyzed by Western blotting and densitometric analysis, as previously reported [14]. Descriptions of these experiments are available in whole online in the Supplementary Material. Samples collected on days zero, 7, and 14, were also analyzed for SerpinA3 by ELISA (EH411RB kit, Thermo Fisher Scientific Inc., Waltham, MA, USA), following the manufacturer’s instructions. Serum concentrations of IL-6, IL-10, and TNF-α were analyzed with commercially available ELISA kits (DY206-05 for IL-6, DY217B-05 for IL-10, and DY210-05 for TNF-α; R&D Systems), following the manufacturer’s instructions.

### 2.2. Gas Chromatography/Mass Spectrometry (GC/MS) Analysis

Metabolomics analysis of serum samples collected on days zero and 3 in all patients and at day 90 in those who survived, was performed by GC/MS. A pool sample for quality control (QC) assessment was generated using equal volumes of every sample included in the metabolomic analysis. The samples were derivatized as previously reported [15]. Briefly, 45 µL was transferred to a microtube and 10 uL of internal standard (tridecanoic acid 0.5 mg/mL) was added. Thereafter, the sample was extracted with 1:4 chloroform-methanol (300 µL), vortexed for 2 min and precipitated for 20 min at −20 °C. The microtube was then centrifuged at 20,000× *g* for 5 min (at 4 °C) to remove proteins and impurities. The supernatant was recovered and dried overnight in a SpeedVac (Savant SPD121P-Thermo Scientific), after which the resulting powder was incubated with 40 µL of methoxyamine diluted in pyridine (20 mg/mL) for 90 min at 37 °C and thereafter derivatized with 40 µL MBSTFA with 1% TMCS for 30 min at room temperature. One microliter of this final mix was injected in an Agilent GC/MS system (Agilent 5977A/7890B, Santa Clara, CA, USA) in splitless mode employing a capillary HP5MS column (30 m × 250 µm × 0.25 µm, Agilent) with a flow of 1 mL/min. An untargeted analysis was performed covering species between 50 and 500 *m*/*z* through electronic ionization of 70 eV. The oven was set by increments of 10 °C/min from 60 °C to 325 °C, with a final step of 10 min holding. Every 6 injections were followed by an injection of the QC sample.

Mzdata archives were obtained with the Chemstation software (Agilent Technologies, Santa Clara, CA, USA; https://www.agilent.com/en/product/software-informatics/analytical-software-suite/chromatography-data-systems/openlab-chemstation, accessed on 8 January 2025) and chromatogram deconvolution and alignment were performed using mzMine3.0 with the following parameters: RT range, 5.5–27.5 min; *m*/*z* range, 50–500; *m*/*z* tolerance, 0.5; noise level, 1 × 103; and peak duration range, 0.01–0.2 min. The rule of 80 was applied, and the peaks were filtered according to a maximum relative standard deviation (RSD) of 30% of the QC sample peaks. For peak identification, the National Institute of Standards and Technology (NIST) 2.0 spectral library with minimal values of >70% confidence and Match and reverse Match > 700 was employed. The peaks below these parameters were omitted.

### 2.3. Operational Definitions

AKI was defined and stratified according to the KDIGO (Kidney Disease: Improving Global Outcomes) guidelines, with the sCr criteria; urinary output was not registered [16]. The mean sCr value 6 months before hospitalization was considered as the baseline sCR or the minimum sCr value obtained during hospitalization if previous values were unavailable [17]. The Charlson comorbidity index was calculated to summarize comorbidity information [18]. Complete renal recovery (CRR) was defined as a sCr value within 25% of the baseline sCr, and partial renal recovery (PRR) was defined as a sCr 25% above the baseline sCr independently of the KRT at 90 days [19]. Registered mortality refers to that which occurred during the COVID-19 hospitalization.

### 2.4. Statistical Analysis

The distribution of continuous variables was evaluated with the Kolmogorov–Smirnov test. Descriptive statistics are expressed as percentages, means (standard deviation), and medians (interquartile range), as appropriate. The Mann–Whitney U test was employed to compare the baseline characteristics between patients with complete renal recovery and those who did not recover renal function at 90 days, as well as alive vs. deceased cases at discharge from the hospital, and the Student’s *t*-test was applied to variables that followed a normal distribution. Categorical variables were analyzed by the Chi-square test or Fisher’s exact test.

Factors associated with CRR and PRR as well as the “alive or deceased” outcome were evaluated by univariate logistic regression analysis. All variables with a probability value < 0.05 and the previously mentioned factors associated with the primary outcome were selected for the multivariate analysis. Age, sex, and Sequential Organ Failure Assessment (SOFA) Score that were significant on the univariate analysis, as well as every biomarker, were examined with a receiving operating characteristic curve (ROC) to analyze precision in CRR. Age, sex, and the PaO_2_/FiO_2_ ratios that were significant, as well as the biomarker data, were analyzed by the ROC for mortality.

The kinetics of uSerpinA3 were analyzed by ANOVA for repeated measures; missing data were imputed with medians and then transformed to decimal logarithms. All statistics were two-tailed and a *p* < 0.05 was considered statistically significant. All analyses were performed using the SPSS 26.0 statistical software (IBM, Armonk, NY, USA, EE. UU.).

For the metabolomics analyses, univariate analysis of the peak heights obtained from deconvolution and alignment were sum-normalized. The Kruskal–Wallis test, Dunn test, and graphing were performed with GraphpadPrism8. The heatmap, hierarchical clustering (Ward method), and principal component analysis (PCA) were computed with Metaboanalyst 3.0 using sum-normalized heights values. For the PLSDA analysis, the raw data were sum-normalized, mean-centered and log-transformed.

## 3. Results

### 3.1. Patient Characteristics and Outcomes

**Figure 1:**
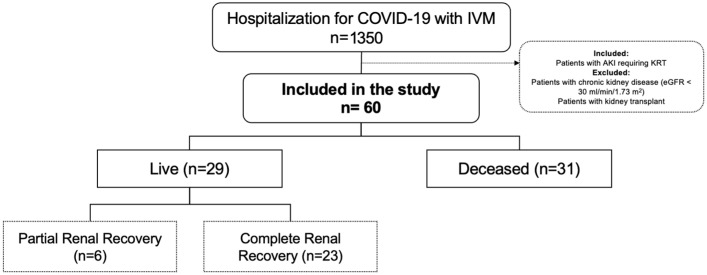
Flowchart of the patients included in the study and their outcome at discharge (Live or deceased). Renal outcome 90 days after the start of KRT (complete and partial renal recovery) is ilustrated in the survivors. Abbreviations: IVM, invasive mechanical ventilation; AKI, actue kidney injury; KRT, kidney replacement therapy; eGFR, estimated glomerular filltration rate.

There were a total of 1350 hospitalizations due to COVID-19 that required IMV during the study period, of whom 60 (4.4%) patients fulfilled the inclusion criteria (Figure 1), All patients included in the present study met the biochemical criteria for initiating renal replacement therapy (RRT); additionally, 80% presented with oliguric acute kidney injury (AKI). Table S1 shows the patients’ clinical and laboratory characteristics upon initiation of KRT. From the total number of patients, 29 (48%) survived hospitalization; there were no statistically significant differences between the live and deceased patients in terms of baseline clinical features, laboratory values upon KRT initiation, severity scores, or administrated drugs, and only the creatine phosphokinase levels were greater in the deceased patients (*p* = 0.023) (Table 1).

All 29 patients who survived hospitalization recovered renal function, 23 (79%) had CRR and the remaining 6 (21%) had PRR at 90 days. The 31 patients who died during hospitalization were all KRT-dependent. The only differences between patients with CRR and those with PRR were the number of leukocytes (*p* = 0.003) and the serum ferritin concentration (*p* = 0.050), (Table 2).

### 3.2. Prediction of Complete Renal Recovery

Comparison of the absolute concentrations of urinary (NGAL, KIM-1, and SerpinA3) and serum (IL-6, IL-10, and TNF-α) biomarkers between CRR and PRR patients, yielded no significant differences at all time points measured (Table S2). To predict CRR, the areas under the curve (AUC) of each serum biomarker at all time points were analyzed; the results are shown in Table 3. In samples at zero time, uKIM-1 exhibited an AUC of 0.71 (95% CI, 0.55–0.86, *p* = 0.017), whereas immunoblotted uSerpinA3 showed post-KRT AUCs of 0.68 (*p* = 0.041) and 0.71 (*p* = 0.030) on days 7 and 14, respectively; at both time points, uSerpinA3 determined by ELISA showed a cutoff value of 6 μg/mg, a sensitivity of 84% with 30% specificity, a 55% positive predictive value, and a 65% negative predictive value. The remaining serum and urinary biomarkers had an AUC between 0.4 and 0.6, with no associated statistically significant differences.

Finally, we analyzed the clinical factors associated with CRR, and a SOFA score > 8 remained significant when the analysis was adjusted for age and sex (Tables S3 and S4). We constructed predictive CRR models by combining clinical variables with the urinary and serum biomarkers obtained upon initiation of KRT. The addition of the biomarkers to the clinical variables slightly improved the performance of some biomarkers: age + uNGAL (AUC 0.67, 95% CI 0.52–0.82, *p* = 0.054), and sex (male) + uNGAL (AUC 0.67, 95% CI 0.52–0.82, *p* = 0.051). The association of three clinical variables with uNGAL did not improve performance. However, the association of sex (male) + uKIM-1 and of SOFA > 8 points + uKIM improved performance vis-á-vis the individual clinical variables (AUC 0.69 and 0.70, respectively). IL-10 improved the performance of age and yielded a trend towards a significant difference when associated with the male sex and a SOFA score > 8. The association of three clinical variables to IL-10 led to an AUC of 0.69, with a 95% CI of 0.55–0.83 (*p* = 0.013). The other models are shown in Table S5. Comparison of the AUC of a biomarker alone vs. the biomarker plus the clinical variables did not lead to a statistically significant result.

### 3.3. Prediction of Mortality

Comparison of the absolute biomarker concentrations between alive and deceased patients revealed differences in uKIM-1 levels on day zero (upon KRT initiation), with a median of 3.63 in the alive patients and 1.77 in those that died (*p* = 0.028) (Table S6). A uKIM-1 value below 1.5 demonstrated a sensitivity of 71%, specificity of 60%, and a negative predictive value of 84% for predicting mortality. There were no differences in the remaining urinary and serum biomarkers at all time points. Upon comparison of the biomarker levels with those in control cases, it was detected that critically ill COVID-19 controls without AKI had lower levels of uSerpinA3, uKIM-1, uNGAL, TNF-α, and IL-10 (Table S6).

The AUC of the biomarkers predicting mortality are shown in Table 4. In the initial sample (i.e. at zero time), uKIM-1 showed an AUC of 0.68 (95% CI 0.53–0.84, *p* = 0.028). The uSerpinA3 measured by Western blotting on days 7 and 14, exhibited an AUC of 0.75 and 0.76, respectively (*p* = 0.007 and 0.015, respectively). Among the serum biomarkers, only IL-10 showed a trend to significance, with an AUC of 0.64, and a *p* value equal to 0.057. There were no significant differences in the remaining biomarkers.

Clinical variables that could be associated with mortality were analyzed by univariate and multivariate analyses (Tables S7 and S8). Rough analyses of patient age, sex, and the PaO_2_/FiO_2_ ratio revealed a tendency towards significance, with an OR of 0.989 per every 0.011 decrease in the quotient; nevertheless, the age- and sex-adjusted analysis was not significant (Table S8).

The performance of the clinical variables as mortality predictors, in conjunction with the studied biomarkers, was then analyzed. Adding patient age to IL-10 improved performance with an AUC of 0.73 (95% CI: 0.60–0.86, *p* = 0.002), and uKIM-1 + sex (male) had an AUC of 0.68 (95% CI 0.53–0.84, *p* = 0.029). The remaining elaborated models did not improve precision (Table S9). Analyzing only the biomarker’s AUC vs. that of the biomarker plus clinical data did not yield a significant difference.

### 3.4. The uSerpinA3 Kinetics and 90-Day Outcome

**Figure 2:**
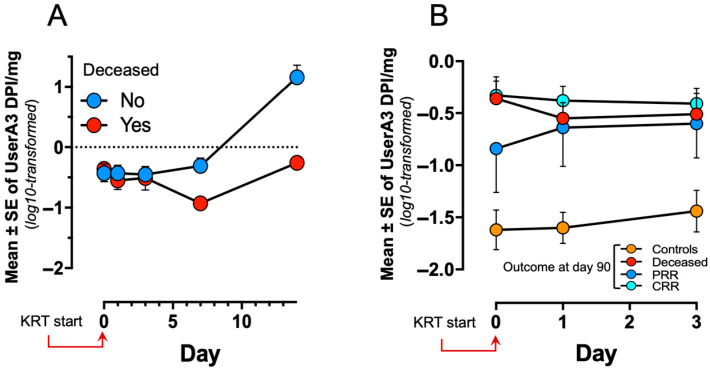
Marginal means in repeated samples of uSerpinA3 according to their outcome at hospital discharge: (**A**) Deceased or not. (**B**) Outcome including controls, deceased, partial renal recovery (PRR) and complete renal recovery (CRR).

The evaluation of uSerpinA3 kinetics between live and deceased cases showed a statistically significant difference (*p* < 0.01) (Figure 2A). Nevertheless, when expression of uSerpinA3 in the controls was analyzed, the latter had lower biomarker marginal means (*p* < 0.01) (Figure 2B).

**Figure 3:**
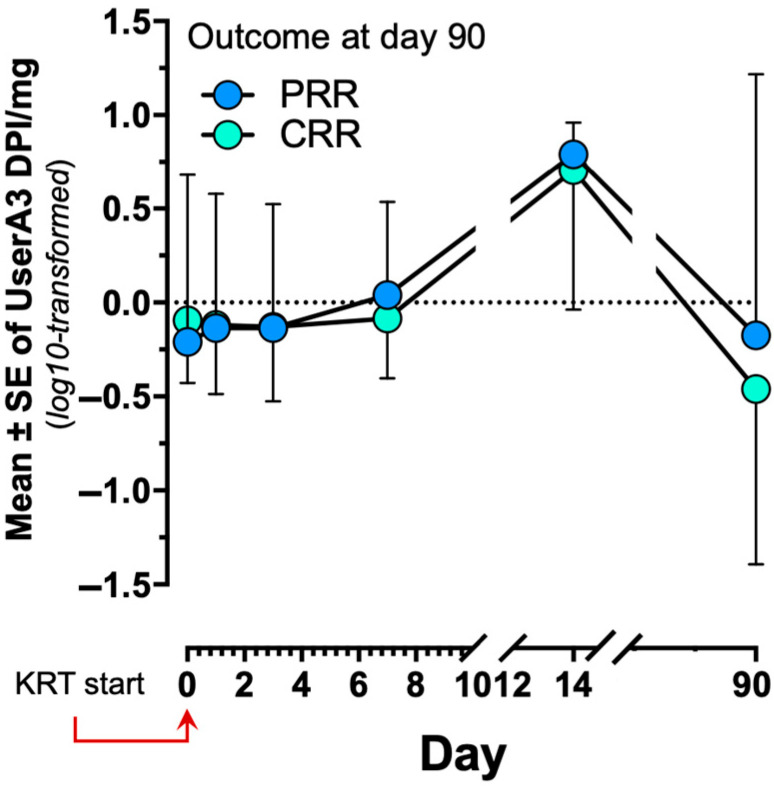
Marginal means in repeated samples of uSerpinA3 according to complete or partial renal recovery at 90 days.

Finally, analysis of the kinetics of uSerpinA3, including those samples obtained on day 90 (survivors), showed that in patients warranting KRT, biomarker levels increased significantly on days 7 and 14, and thereafter markedly decreased on day 90, with figures comparable to those observed in the control group without AKI (Figure 3).

### 3.5. Metabolomics Results

Thirty-three different metabolites were included in the final analysis. The metabolites were enriched with Krebs cycle intermediaries, amino acids and derivatives, fatty acids, and sugars. The robustness of the method was estimated using principal component analysis (PCA). This unsupervised method showed a low variability between the injections of QC samples, which is depicted as a compact well-defined grouping in Figure S1.

**Figure 4:**
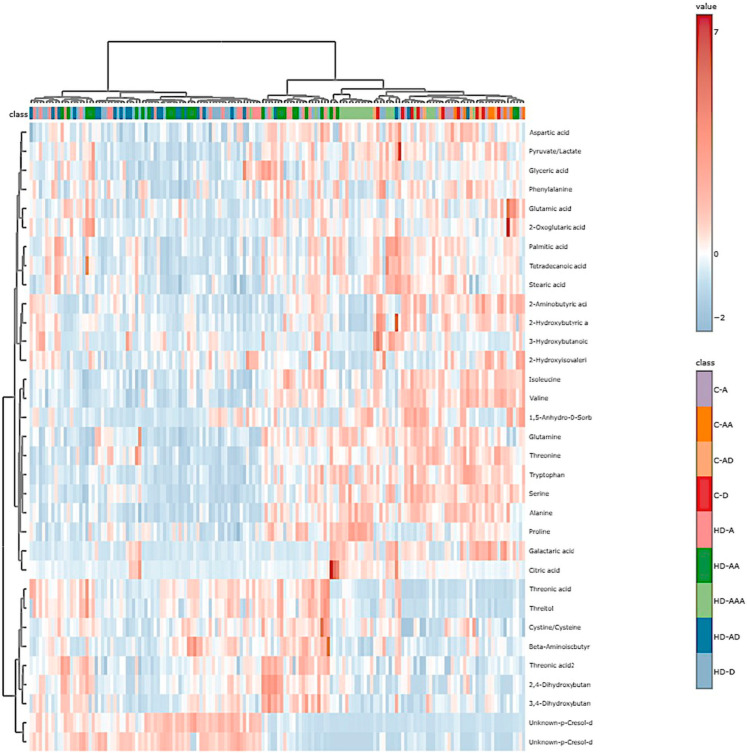
Heatmap and hierarchical clustering analysis of the metabolic profiles of groups C-A (Initial sample surviving controls), C-AA (third sample surviving controls), C-D (initial sample dead controls), C-AD (third sample dead controls), HD-A (initial sample surviving hemodialysis patient), HD-AA (third sample surviving hemodialysis patient), HD-D (Initial sample dead hemodialysis patient), HD-AD (third sample dead hemodialysis patient), and HD-AAA (full recovery group).

To globally explore the metabolomic changes in our cohort, a heat map with hierarchical clustering analysis was constructed (Figure 4). Patients under hemodialysis (HD) segregate well from the non-hemodialyzed (C) and fully recovered (HD-AAA) groups, while final outcome (death or survival) did not segregate homogeneously when employing this analysis.

**Figure 5:**
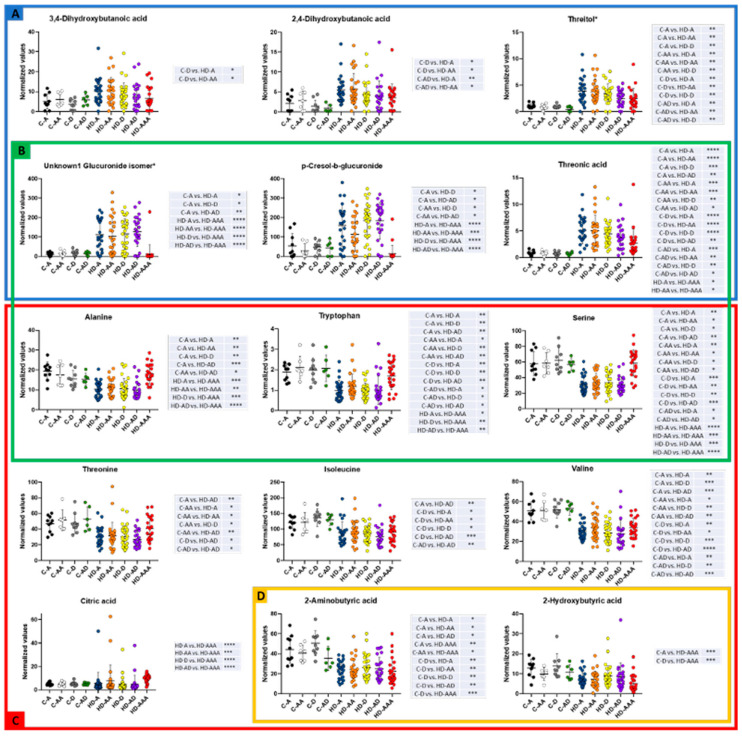
Changes in the metabolite’s levels between groups C-A (initial sample surviving controls), C-AA (third sample surviving controls), C-D (Initial sample dead controls), C-AD (third sample dead controls), HD-A (initial sample surviving hemodialysis patient), HD-AA (third sample surviving hemodialysis patient), HD-D (initial sample dead hemodialysis patient), HD-AD (third sample dead hemodialysis patient), and HD-AAA (full recovery group). The graphs were constructed with normalized values and statistically analyzed employing the Kruskal-Wallis and Dunn tests. Significance of values: * *p* < 0.05, ** *p* < 0.01, *** *p* < 0.001, **** *p* < 0.0001. (**A**) metabolites that increased in the hemodialyzed group when compared with non-hemodialyzed patients; (**B**) those metabolites whose levels changed only in the hemodialyzed group and that are probably related to this condition since levels were different in the full recovery patients (HD-AAA); (**C**) metabolites that decreased in the hemodialysis groups when compared with non-hemodialyzed patients; (**D**) metabolites that were high in patients with COVID-19 without renal failure.

Figure 5 shows those metabolites that changed significantly between the groups included in the final cohort. Panel A shows those metabolites that increased in the hemodialyzed group when compared with non-hemodialyzed patients, while panel B shows those metabolites whose levels changed only in the hemodialyzed group and that are probably related to this condition since levels were different in the full recovery patients (HD-AAA). Panel C presents those metabolites that decreased in the hemodialysis groups when compared with non-hemodialyzed patients. Finally, panel D shows those metabolites that were high in patients with COVID-19 without renal failure and that were low in hemodialyzed and/or full disease recovery groups.

**Figure 6:**
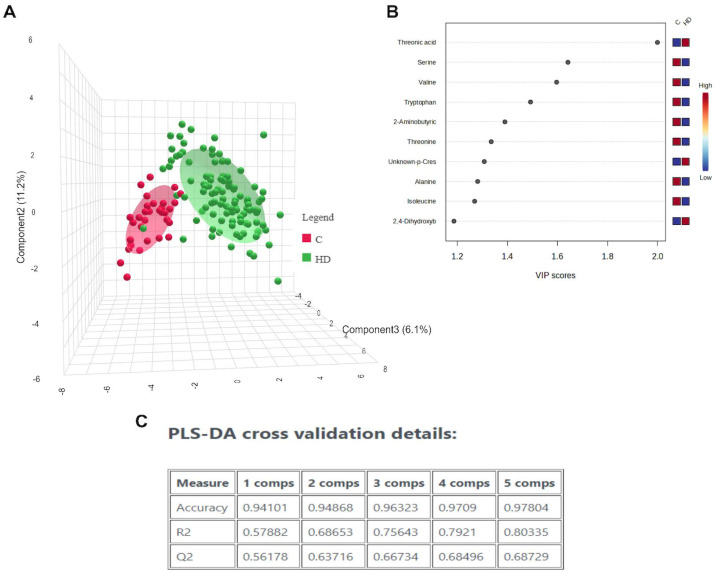
(**A**) PLS-DA of COVID vs COVID+hemodialyzed (HD) patients, independent of blood sample collection or fatal outcome. The analysis was generated using the peak heights after sum normalization, mean centering and log transformation. (**B**) Variable importance of projection (VIP) of the top ten metabolites; intensity gradient is shwed in the right vertical colored bar. (**C**) Results of cross validation. R2: goodness of fit. Q2: predictive ability.

Patients with decreased renal function exhibited a distinct metabolomic profile. Employing multivariate supervised analysis (PLS-DA) on all groups, we identified that the recovery of patients belonging to the HD-AAA groups, was different than in COVID-19 patients, despite the KRT needs (Figure S2). The main metabolic changes in patients with COVID-19 vs. those with COVID-19 that required KRT (excluding those patients who had died at the time of sample collection) are summarized in Figure 6. Variable Importance in Projection (VIP) analysis of metabolites revealed differences in several amino acids (serine, valine, threonine, alanine, isoleucine, and tryptophan), threonic acid, 2-aminobutyric acid, and an unknown p-cresol derivative between COVID-19 and COVID-19 + KRT groups. These metabolites seem to be associated with better kidney function in COVID-19 patients. On the other hand, considering the patients fate (live or dead) and need of KRT, this statistical method employed did not allow us to clearly identify differences between the groups, thus suggesting that the resolution of the GC/MS approach did not detect clear and significant differences in the metabolomic profiles of these particular conditions (Figure S3). In the same vein, employing the same supervised analysis on hemodialyzed or non-hemodialyzed patients but including separately the final outcome (death or alive) as a grouping feature, there were no clear metabolomic differences that could yield a predictor of a fatal outcome in COVID-19 (Figure S4) or in COVID-19-HD patients (Figure S5).

## 4. Discussion

This study revealed that, among all the studied biomarkers, uSerpinA3 and uKIM-1 exhibited the best performance as predictors of complete renal function recovery and mortality in patients with COVID-19 associated with AKI and KRT. uKIM-1 was an early biomarker at time zero, whereas uSerpinA3 was a late-onset biomarker (at 7 and 14 days) useful for predicting CRR and mortality in that particular population. This is the first study to demonstrate an association of these urinary biomarkers with renal recovery in a critically ill COVID-19 population.

SerpinA3 has been associated with signaling in a number of pathways pertaining to pro- and antifibrotic balance (e.g. vascular endothelial growth factor, connective tissue growth factor, and WNT/β-catenin pathways in different animal models) [12,13]. Based on previous studies, we speculated that SerpinA3 is expressed and secreted by tubular epithelial cells in response to an inflammatory milieu resulting from severe tubular injury [13]. In fact, the present study documented the increase in uSerpinA3 throughout the follow-up of all patients, leading to the theory that increased uSerpinA3 excretion may reflect the established repair/inflammation mechanisms. Nevertheless, after 90 days, we observed that the uSerpinA3 excretion became comparable to that in controls with no tubular injury (Figure 3 and Figure 4); this could suggest that the repair/tubular injury process had ceased. This finding correlates with the time that appears to be required to recover renal function after an AKI event, as observed by both previous reports and the present study [16,20,21].

We previously studied the kinetics of uSerpinA3 in a cohort of 60 patients with class III and class IV lupus nephritis (LN), finding greater uSerpinA3 concentrations in class IV LN patients, probably as a result of a greater degree of disease-mediated activity and inflammation. When the uSerpinA3 behavior was analyzed according to the follow-up of the treatment response, the patients with no treatment response or with a partial response showed greater urinary uSerpinA3 levels compared with full treatment responders [14]. This was probably the result of an unresolved inflammatory microenvironment that enabled distinguishing between active LN and chronic kidney scarring; this possibility agrees with the hypothesis generated in the present study, in which the increase in uSerpinA3 in critically ill COVID-19 patients that developed AKI was secondary to the inflammatory state at the tubular level.

The uSerpinA3 was compared with other tubular injury biomarkers that have been widely reported, such as uKIM-1 and uNGAL. In patients with COVID-19, KIM-1 has particularly been used to predict AKI of any degree [22], as well as KRT requirement and death [23]. Although a number of studies have previously documented that an AUC between 0.5 and 0.8 can predict these outcomes, none have evaluated renal function recovery after an AKI episode requiring KRT. uNGAL has also been studied in this population and predicted the same outcome as uKIM-1 in terms of AUC [24,25,26]. It is noteworthy that, to date, previous studies have documented the occurrence of higher levels of uKIM-1 and uNGAL with fatal outcomes [22,27,28]. Nonetheless, ∼50% of patients in those studies were on IMV and only 12–15% had AKI. Our patients encompassed a more severe disease spectrum, so perhaps it was the respiratory compromise that mediated the dire outcomes.

Several clinical and demographic variables, such as greater age, multiple comorbidities (hypertension, diabetes mellitus, cardiovascular disease, CKD), the type and severity of the acute disease leading to the AKI episode, and the duration of AKI, among others, have been considered as factors associated with lower renal function recovery and mortality [8,29]. The most studied clinical variable employed to predict KRT weaning and kidney function recovery is urinary volume, with a good predictive power (AUC 0.81) [30]. This is why we developed constructs to predict CRR and mortality by adding biomarkers, biologically plausible clinical variables, and the significant variables detected in the multivariate analysis. As shown in Tables S5 and S9, the performance is not superior in the proposed models in comparison with the biomarkers alone. Unlike other reports, the urinary volume in our patients was not important as a predictor of renal recovery.

Particularly at the beginning of the COVID-19 pandemic, the inflammatory state in AKI patients was considered a key pillar of the disease’s pathogenesis, which explains why various cytokine profiles were used to predict adverse outcomes and even considered as treatment targets [31,32]. In our study, we measured IL-6, IL-10, and TNF-α, and none of these markers performed well as predictors of the study outcomes.

In patients with AKI and COVID-19, the kidney function recovery previously reported reaches up to 80%, depending on the included patients and the applied definitions [5,6,7]. In our study, all surviving patients were dialysis-free at 90 days. However, patients with an eGFR < 30 mL/min, in whom baseline kidney injury was clearly associated with a limited recovery after a severe AKI episode, were excluded, which may partially explain the high recovery rates observed.

According to the present results, uSerpinA3 is particularly associated with mortality; surviving patients initially had similar biomarker levels, perhaps as an inflammatory response and superior cellular repair. To date, no similar findings have been reported, but notably, employing proteomic approaches, SerpinA3 has been associated with disease severity [13,14]. A cohort of 71 patients (20 controls, 19 with non-critical COVID-19, and 33 with critical COVID-19) was studied in India [33], and the authors found higher serum SerpinA3 in patients with critical COVID-19, as a surrogate of disease severity. In the COVIDomics study, elevated SerpinA3 levels were also documented in more severely ill patients at greater risk of disease progression [34], although they were not associated with mortality. Although neither SerpinA3 levels in blood nor a different spectrum of COVID-19 patients were included in the present study, it is known that these factors are closely linked to systemic inflammatory states. In fact, higher levels of SerpinA3 in urine among survivors and in patients with tubular injury were observed. Although we are unaware of this biomarker’s behavior in blood, we can speculate that, based on the included population’s disease severity, the concentration of this marker was probably elevated. Further, the behavior of SerpinA3 in blood may not reflect the events occurring in kidney tissue as a result of AKI.

Regarding the metabolomics analysis, although we identified four distinct profiles, the metabolite that maintained statistical significance in predicting mortality was p-cresol glucuronide. Several metabolomics studies have examined blood or urine to identify metabolite changes associated with CKD or to identify metabolite predictors of CKD progression and mortality. To date, no metabolite has emerged as an unequivocal causal factor in the pathogenesis of CKD or its complications, which is probably due to the heterogeneity of the study populations and also to the fact that the large number of metabolomic perturbations that manifest as renal clearance is lost [35]. However, an important issue that has currently emerged is the fundamental contribution of the gut microbiome on the blood metabolome in CKD. For example, Meyer et al. [36] used blood from nine patients under hemodialysis treatment with intact colons and six hemodialysis patients who had previously undergone colectomy. Compared with end stage kidney disease (ESKD) patients with intact colons, those who had undergone colectomy exhibited markedly lower levels of at least 35 metabolites, including indoxyl sulfate and p-cresol sulfate. Moreover, the levels of these colon-derived metabolites were substantially higher in ESKD patients than in individuals with normal renal function, and most were not effectively removed by conventional hemodialysis.

On the other hand, we observed that among the other metabolic pathways affected were those related to amino acid metabolism. In controls, we detected significant increases in those amino acids that result from muscle protein catabolism, which is quite common in severely ill patients [35]. The breakdown of muscle proteins provides the amino acids needed to compensate for insufficient dietary protein intake and depleted protein stores [37]. In the present study, patients on hemodialysis presented lower levels of amino acids compared to controls; although both populations were critically ill, the controls were not under RRT, and the samples analyzed in those with RRT were obtained several days after their critical condition, once they were required to start RRT. This might explain the lower amino acid levels detected in the latter patient group, which was probably due to depletion of the amino acid reserve. We also observed a different behavior in 2-aminobutyric acid and 2-hydroxybutyric acid, which remained at lower levels in those who required RRT, regardless of whether they survived compared to controls. Both metabolites are involved in oxidative stress, and it has been documented that levels of 2-aminobutyric acid reflect a compensation of glutathione against cellular stress [38]. In this vein, it is well known that glutathione depletion directly correlates with ischemia–reperfusion, which is a common cause of acute kidney injury [39].

Some limitations of our study should be recognized. First, measurement of SerpinA3 was semi-quantitative (by Western blot in all cases), and although ELISA was employed on day zero (KRT initiation) and on weeks 1 and 2, this technique has not been fully standardized, and there may be considerable variation between manufacturers and even between batches of the same kit. Nevertheless, Western blot results might overcome this limitation, as demonstrated in our previous studies [13,14]. Second, this study included a relatively a small sample size due to restrictions imposed by our inclusion and exclusion criteria. Nonetheless, the greatest strength of the study lies in the fact that an extended kinetics profile of biomarkers was obtained in all patients. It should be recognized that the present findings require validation at a greater scale and in populations with different AKI etiologies, given the impact of the long-term repercussions in renal recovery.

## 5. Conclusions

Urinary SerpinA3 is a late-onset biomarker useful for predicting the recovery of renal function as well as mortality. uKIM-1 is an early-onset biomarker, capable of predicting the same outcomes in patients with critical COVID-19 and AKI requiring KRT. A SOFA score > 8 and a decrease in the PaO_2_/FiO_2_ ratio were the main clinical risk factors associated with renal recovery and mortality, respectively. When adding these clinical variables to the studied biomarkers, the predictive performance of the studied outcomes does not improve. Metabolic analysis appears promising for identifying affected pathways and their clinical impact; however, given the number of patients and inclusion criteria, generalization of the findings to other populations is necessary.

## Figures and Tables

**Table 1:** Clinical characteristics of patients at KRT initiation, divided according to the outcome of hospitalization live vs. deceased.

	Live (*n* = 29)	Deceased (*n* = 31)	*p*-Value
Demographics			
Age, years	51 ± 12.2	56 ± 12.2	0.085
Male, *n* (%)	20 (69)	26 (84)	0.173
Body mass index, kg/m^2^	31 (29–35)	29 (27–38)	0.240
Charlson index	1 (0–2)	2 (0–3)	0.323
SOFA score	10 (9–11)	10 (9–11)	0.542
Days of hospitalization at the beginning KRT	5 (2–12)	6 (4–12)	0.418
Days of IMV at the beginning KRT	3 (2–5)	6 (3–9)	0.079
**Kidney function**			
Baseline SCr, mg/dL	1 (0.8–1.2)	1 (0.9–1.2)	0.823
SCr at KRT initiation, mg/dL	5.1 (3.5–6)	4.2 (3.3–5)	0.162
Urine output at KRT initiation, mL	612 (280–1347)	994 (250–2112)	0.416
**Laboratory at KRT initiation**			
Leukocytes, x 1000/mm^3^	12 (9–15)	12 (8–17)	0.784
C-reactive protein, mg/dL	16.2 (10.3–27)	19 (7.6–29.3)	0.906
Creatine kinase, U/L	369 (69–1049)	1013 (302–1852)	0.023
Lactate dehydrogenase, U/L	433 (313–552)	482 (335–586)	0.608
Ferritin, ng/mL	939 (532–1689)	1202 (613–1855)	0.276
PaO_2_/FiO_2_ ratio	144 (113–170)	123 (96–154)	0.141
**Treatments in ICU at KRT initiation**			
Carbapenems, *n* (%)	18 (62)	20 (65)	0.844
Vancomycin, *n* (%)	13 (45)	14 (45)	0.979
Antifungal therapy, *n* (%)	3 (10)	3 (10)	1.000
Norepinephrine, *n* (%)	22 (76)	27 (87)	0.327

Note. Continuous variables are expressed as median (interquartile range) or mean (standard deviation). Abbreviations. SOFA, Sequential Organ Failure Assessment score; KRT, kidney replacement therapy; IMV, invasive mechanical ventilation; SCr, serum creatinine; ICU, intensive care unit; PaO_2_/FiO_2_ ratio, ratio of arterial oxygen partial pressure to fractional inspired oxygen.

**Table 2:** Clinical characteristics of patients included in this study by CRR and PRR.

	CRR (*n* = 23)	PRR (*n* = 6)	*p*-Value
Demographics			
Age, years	51 ± 13.4	47 ± 9.8	0.448
Male, *n* (%)	16 (70)	4 (67)	1.000
Body mass index, kg/m^2^	31.2 (28.9–36.6)	31.2 (31.1–33.4)	0.860
Charlson index, *n* (%)	1 (0–3)	2 (0–4)	0.494
SOFA score	10 (9–11)	9 (7–12)	0.525
Days of hospitalization at the beginning KRT	6 (2–15)	5 (4–8)	0.733
Days of IMV at the beginning KRT	3 (2–8)	3 (2–5)	0.581
**Kidney function**			
Baseline SCr, mg/dL	1 (0.8–1.2)	0.9 (0.7–1.1)	0.796
SCr at KRT initiation, mg/dL	4.5 (3.3–5.9)	5.7 (5.1–6.9)	0.321
Urine output at KRT initiation, mL	587 (301–1316)	1091 (249–1855)	0.561
SCr at discharge, mg/dL	0.83 (0.68–1.76)	3.89 (1.85–4.87)	0.003
**Laboratory at KRT initiation**			
Leukocytes, ×1000/mm^3^	13 (10–16)	7 (6–9)	0.003
C-reactive protein, mg/dL	19.2 (10.2–28.3)	12.3 (10.4–18.4)	0.527
Creatine kinase, U/L	340 (100–1225)	267 (43–753)	0.251
Lactate dehydrogenase, U/L	468 (347–554)	312 (275–524)	0.212
Ferritin, ng/mL	1171 (602–1815)	443 (327–901)	0.050
PaO_2_/FiO_2_ ratio	144 (110–170)	160 (113–175)	0.667
**Treatments in ICU at KRT initiation**			
Carbapenems, *n* (%)	17 (74)	2 (33)	0.156
Vancomycin, *n* (%)	11 (48)	3 (50)	1.000
Antifungal therapy, *n* (%)	3 (13)	1 (17)	1.000
Norepinephrine, *n* (%)	20 (87)	3 (50)	0.120

Note. Continuous variables are expressed as median (interquartile range) or mean (standard deviation). Abbreviations. CRR, complete renal recovery; PRR, partial renal recovery; SOFA, Sequential Organ Failure Assessment score; KRT, kidney replacement therapy; IMV, invasive mechanical ventilation; SCr, serum creatinine; ICU, intensive care unit; PaO_2_/FiO_2_ ratio, ratio of arterial oxygen partial pressure to fractional inspired oxygen.

**Table 3:** Area under the receiver-operating characteristics curve of biomarkers for predicting complete renal recovery from AKI with KRT.

Biomarker	Time Point	AUC (95% CI)	*p*-Value
Urine biomarker			
KIM-1, µg/mg	Day 0	0.71 (0.55–0.86)	0.017
	Day 1	0.63 (0.46–0.81)	0.143
	Day 3	0.54 (0.37–0.71)	0.673
	Day 7	0.52 (0.34–0.70)	0.846
	Day 14	0.60 (0.40–0.80)	0.283
NGAL, µg/mg	Day 0	0.67 (0.52–0.82)	0.051
	Day 1	0.55 (0.37–0.72)	0.626
	Day 3	0.50 (0.32–0.67)	0.952
	Day 7	0.55 (0.37–0.73)	0.576
	Day 14	0.51 (0.32–0.70)	0.927
SerpinA3 *, DPI/mg	Day 0	0.59 (0.43–0.75)	0.300
	Day 1	0.61 (0.45–0.78)	0.223
	Day 3	0.56 (0.39–0.73)	0.492
	Day 7	0.68 (0.52–0.85)	0.041
	Day 14	0.71 (0.53–0.89)	0.030
SerpinA3 **, µg/mg	Day 0	0.61 (0.47–0.76)	0.140
	Day 7	0.49 (0.31–0.66)	0.865
	Day 14	0.58 (0.39–0.77)	0.425
Urine output > 500 mL	Day 0	0.53 (0.38–0.68)	0.715
Urine output > 1 L	Day 0	0.61 (0.47–0.76)	0.144
**Plasma biomarker**			
IL-6, pg/mL	Day 0	0.58 (0.37–0.67)	0.830
IL-10, pg/mL	Day 0	0.64 (0.50–0.79)	0.072
TNF-alpha, pg/mL	Day 0	0.57 (0.41–0.73)	0.366

Abbreviations. 95% CI, confidence interval at 95%; AUC, area under the curve. * Using Western blot. ** Using ELISA.

**Table 4:** Area under the receiver-operating characteristics curve of biomarkers for predicting mortality.

Biomarker	Time Point	AUC (95% CI)	*p*-Value
**Urine biomarker**			
KIM-1, µg/mg	Day 0	0.68 (0.53–0.84)	0.028
	Day 1	0.60 (0.43–0.77)	0.254
	Day 3	0.51 (0.33–0.69)	0.925
	Day 7	0.54 (0.35–0.73)	0.702
	Day 14	0.56 (0.34–0.79)	0.561
NGAL, µg/mg	Day 0	0.63 (0.47–0.79)	0.123
	Day 1	0.51 (0.33–0.68)	0.934
	Day 3	0.43 (0.26–0.61)	0.439
	Day 7	0.51 (0.31–0.71)	0.959
	Day 14	0.56 (0.35–0.77)	0.561
SerpinA3 *, DPI/mg	Day 0	0.52 (0.36–0.69)	0.779
	Day 1	0.58 (0.41–0.75)	0.366
	Day 3	0.54 (0.37–0.72)	0.622
	Day 7	0.75 (0.59–0.92)	0.007
	Day 14	0.76 (0.58–0.95)	0.015
SerpinA3 **, µg/mg	Day 0	0.64 (0.48–0.80)	0.097
	Day 7	0.54 (0.37–0.72)	0.646
	Day 14	0.47 (0.26–0.68)	0.758
Urine output > 500 mL	Day 0	0.51 (0.37–0.66)	0.859
Urine output >1 L	Day 0	0.59 (0.44–0.73)	0.249
**Plasma biomarker**			
IL-6, pg/mL	Day 0	0.57 (0.43–0.72)	0.311
IL-10, pg/mL	Day 0	0.64 (0.50–0.79)	0.057
TNF-alpha, pg/mL	Day 0	0.52 (0.37–0.67)	0.830

Abbreviations. 95% CI, confidence interval at 95%; AUC, area under the curve. * Using Western blot. ** Using ELISA.

## Data Availability

The datasets used and/or analyzed during the current study are available from the corresponding author on reasonable request.

## Supplementary Materials

The following supporting information can be downloaded at https://www.mdpi.com/article/10.3390/diagnostics15151960/s1, Table S1: Clinical characteristics of patients included in this study at the beginning of KRT; Table S2: Values of the different biomarkers in patients with CRR and PRR; Table S3: Univariate logistic regression analysis for CRR; Table S4: Univariate and multivariate logistic regression analysis for CRR; Table S5: Area under the receiver-operating characteristics curve of models (clinical variables and biomarkers) for predicting complete renal recovery from AKI with KRT; Table S6: Values of the different biomarkers at the start of KRT in live vs. deceased patients; Table S7: Univariate logistic regression analysis for mortality; Table S8: Univariate and multivariate logistic regression analysis for mortality; Table S9: Area under the receiver-operating characteristics curve of models (clinical variables and biomarkers) for predicting mortality; Figure S1: Principal component analysis of all the patients included in the final analysis; Figure S2: (A) PLS-DA of all the patients included in the final analysis. The analysis was generated using the peak heights after sum normalization, mean centering and log transformation. (B) Variable importance of projection (VIP) of the top ten metabolites. Intensity gradient is shown in the right vertical colored bar. (C) Results of cross validation. R2: goodness of fit. Q2: predictive ability; Figure S3: (A) PLS-DA of COVID patients considering only the KRT and fate (deceased or alive). The analysis was generated using the peak heights after sum normalization, mean centering and log transformation. (B) Variable importance of projection (VIP) of the top ten metabolites. Intensity gradient is shown in the right vertical colored bar. (C) Results of cross validation. R2: goodness of fit. Q2: predictive ability; Figure S4: (A) PLS-DA of COVID patients only considering fate (deceased or alive). The analysis was generated using the peak heights after sum normalization, mean centering and log transformation. (B) Variable importance of projection (VIP) of the top ten metabolites. Intensity gradient is represented by the right vertical colored bar. (C) Results of cross validation. R2: goodness of fit. Q2: predictive ability; Figure S5: (A) PLS-DA of hemodialyzed patients considering fate (deceased or alive). The analysis was generated using the peak heights after sum normalization, mean centering and log transformation. (B) Variable importance of projection (VIP) of the top ten metabolites. Intensity gradient is represented by the right vertical colored bar. (C) Results of cross validation. R2: goodness of fit. Q2: predictive ability.
