# Supplementary material for: Can Biomarkers Predict Kidney Function Recovery and Mortality in Patients with Critical COVID-19 and Acute Kidney Injury?
Source: Diagnostics (Basel). 2025 Aug 5;15(15):1960. doi: 10.3390/diagnostics15151960 (PMC12346628; doi:10.3390/diagnostics15151960)
Supplement: Supplementary file 1 [file diagnostics-15-01960-s001.zip › diagnostics-3708634-supplementary.pdf]

Table S1. Clinical characteristics of patients included in this study at the beginning of KRT.

	All patients (n=60)
**Demographics**	
Age, years	54 ± 12.3
Male, n (%)	46 (77)
Body mass index, kg/m^2	30 (27-35)
Charlson index	2 (0-3)
SOFA score	10 (9-11)
Days of hospitalization at the beginning KRT	6 (3-12)
Days of IMV at the beginning KRT	4 (2-8)
**Kidney function**	
Baseline SCr, mg/dL	1 (0.9-1.2)
SCr at KRT initiation, mg/dL	4.4 (3.3-5.8)
Urine output 24 hrs prior to the KRT initiation, ml	680 (253-1804)
SCr at discharge, mg/dL	1.30 0.73-2.27)
**Laboratory at KRT initiation**	
Leukocytes, x 1000/mm^3	12 (8-15)
C-reactive protein, mg/dL	16.9 (10.2-28.8)
Creatine kinase, U/L	864 (122-1552)
Lactate dehydrogenase, U/L	468 (330-554)
Ferritin, ng/mL	1037 (572-1801)
PaO_2/FiO_2 ratio	129 (101-165)
**Treatments in ICU at KRT initiation**	
Carbapenems, n (%)	38 (63)
Vancomycin, n (%)	27 (45)
Antifungal therapy, n (%)	6 (10)
Norepinephrine, n (%)	49 (82)

Note. Continuous variables are expressed as median (interquartile range) or mean (standard deviation).

Abbreviations. SOFA, Sequential Organ Failure Assessment score; SCr, serum creatinine; KRT, kidney replacement therapy; PaO_2/FiO_2 ratio, ratio of arterial oxygen partial pressure to fractional inspired oxygen; ICU, intensive care unit.

Table S2. Values of the different biomarkers in patients with CRR and PRR.

Biomarker	Time point	CRR (n=23)	PRR (n=6)	*p*-Value
SerpinA3, µg/mg	Day 0	16.62 (3.84-29.44)	9.72 (1.23-77.62)	0.979
	Day 7	10.51 (2.18-27.19)	40.03 (3.97-62.20)	0.467
	Day 14	4.77 (2.14-26.07)	28.43 (1.73-64.52)	0.414
KIM-1, µg/mg	Day 0	3.66 (2.12-8.19)	2.17 (0.65-4.90)	0.333
	Day 1	3.67 (1.56-6.17)	0.78 (0.64-7.46)	0.409
	Day 3	2.97 (1.65-4.77)	0.96 (0.73-5.71)	0.557
	Day 7	2.43 (1.73-3.83)	3.06 (0.74-4.87)	0.867
	Day 14	3.67 (3.22-5.13)	2.15 (1.31-7.81)	0.351
NGAL, µg/mg	Day 0	2108 (1208-5170)	398 (375-3218)	0.264
	Day 1	2726 (399-4217)	530 (499-953)	0.409
	Day 3	1826 (136-3888)	270 (222-683)	0.411
	Day 7	1656 (262-4125)	965 (196-3291)	0.433
	Day 14	1145 (110-3917)	816 (571-2103)	0.726
TNF-alpha, pg/ml	Day 0	33.50 (26.60-52.80)	42.55 (40.30-44.60)	0.212
IL-6, pg/ml	Day 0	75.20 (30.20-112.40)	94.65 (43.00-242.60)	0.477
IL-10, pg/ml	Day 0	23.90 (18.20-39.10)	22.65 (21.80-52.80)	0.694

Table S3. Univariate logistic regression analysis for CRR.

Characteristic	Univariate		
	OR	95% CI	*p*-Value
Age, per year	1.031	0.955-1.111	0.436
Male, vs female	1.214	0.179-8.217	0.842
Body mass index, per kg/m^2	0.994	0.895-1.103	0.907
Charlson index, per point	1.184	0.815-1.719	0.376
SOFA score >8, vs not	11.00	1.271-95.178	0.029
Ferritina ≥500, vs not	5.000	0.728-34.345	0.102
Use of vasopressor, vs not	4.857	0.718-32.867	0.102
Dexamethasone therapy, vs not	0.846	0.141-5.070	0.855

Table S4. Univariate and multivariate logistic regression analysis for CRR.

Characteristic	Univariate			Multivariate		
	OR	95% CI	*p*-Value	OR	95% CI	*p-V*alue
Age, per year	1.031	0.955-1.111	0.436	1.127	0.108-11.725	0.920
Male, vs female	1.214	0.179-8.217	0.842	1.020	0.933-1.114	0.669
SOFA >8, Vs no	11	1.271-95.178	0.029	17.287	1.435-208.23	0.025

Abbreviations. OR, odds ratio; 95%CI, confidence interval at 95%; BMI, body mass index; CKD, chronic kidney disease; ICU, intensive care unit; LDH, lactate dehydrogenase; SOFA, Sequential Organ Failure Assessment score.

Table S5. Area under the receiver-operating characteristics curve of models (clinical variables and biomarkers) for predicting complete renal recovery from AKI with KRT.

Complete renal recovery		AUC (95% CI)	*p*-Value
**Clinical variables**			
Age, per year		0.56 (0.41-0.72)	0.407
Male, vs female		0.56 (0.41-0.71)	0.456
SOFA >8 points at RRT admission		0.55 (0.40-0.70)	0.508
**MODELS***			
Age + KIM-1	Day 0	0.56 (0.40-0.73)	0.481
Age + NGAL		0.67 (0.52-0.82)	0.054
Age + SerpinA3*		0.49 (0.32-0.66)	0.917
Age + SerpinA3**		0.38 (0.22-0.53)	0.154
Age + IL-6		0.60 (0.45-0.74)	0.204
Age + IL-10		0.69 (0.55-0.83)	0.014
Age + TNF-alpha		0.56 (0.41-0.72)	0.407
Age + SerpinA3*	Day 7	0.48 (0.32-0.66)	0.808
Age + SerpinA3*	Day 14	0.46 (0.27-0.66)	0.713
Male + KIM-1	Day 0	0.69 (0.53-0.85)	0.028
Male + NGAL		0.67 (0.52-0.82)	0.051
Male + SerpinA3*		0.64 (0.48-0.80)	0.106
Male + SerpinA3**		0.52 (0.34-0.69)	0.852
Male + IL-6		0.60 (0.45-0.75)	0.199
Male + IL-10		0.64 (0.50-0.79)	0.067
Male + TNF-alpha		0.57 (0.41-0.73)	0.370
Male + SerpinA3*	Day 7	0.52 (0.34-0.69)	0.846
Male + SerpinA3*	Day 14	0.58 (0.39-0.77)	0.391

SOFA >8 points + KIM-1	Day 0	0.70 (0.55-0.86)	0.019
SOFA >8 points + NGAL		0.67 (0.52-0.82)	0.051
SOFA >8 points + SerpinA3*		0.64 (0.48-0.79)	0.120
SOFA >8 points + SerpinA3**		0.54 (0.37-0.70)	0.678
SOFA >8 points + IL-6		0.40 (0.26-0.55)	0.204
SOFA >8 points + IL-10		0.64 (0.49-0.78)	0.082
SOFA >8 points + TNF-alpha		0.57 (0.41-0.73)	0.374
SOFA >8 points + SerpinA3*	Day 7	0.52 (0.34-0.69)	0.846
SOFA >8 points + SerpinA3*	Day 14	0.58 (0.39-0.77)	0.391
Age + Male + SOFA + IL-10	Day 0	0.69 (0.55-0.83)	0.013
Age + Male + SOFA + NGAL		0.67 (0.52-0.82)	0.054
Male + SOFA + KIM-1		0.68 (0.52-0.84)	0.034

***ELISA, **Western blot**

Table S6. Values of the different biomarkers at the start of KRT in live vs deceased patients.

Biomarker	Time point	Controls (n=17)	Live (n=29)	Deceased (n=31)	*p*-Value
SerpinA3, µg/mg	Day 0	0.03 (0.01-0.09)	12.55 (3.24-36.41)	28.12 (9.37-50.28)	0.000/0.097*
	Day 7	0.02 (0.007-0.10)	11.98 (2.46-52.62)	7.43 (2.96-25.91)	0.000/0.646*
	Day 14	0.03 (0.006-0.15)	8.53 (1.73-40.92)	5.90 (3.81-55.39)	0.000/0.758*
KIM-1, µg/mg	Day 0	2.00 (0.88-3.27)	3.63 (1.51-8.19)	1.77 (0.69-2.68)	0.052/**0.028***
	Day 1	-	3.55 (0.85-6.75)	2.02 (1.10-4.24)	/0.254*
	Day 3	-	2.90 (0.96-5.30)	2.30 (0.68-8.09)	/0.925*
	Day 7	1.48 (0.76-3.11)	2.43 (1.51-4.11)	2.31 (0.75-4.72)	0.375/0.702*
	Day 14	1.76 (0.89-3.25)	3.56 (2.12-5.49)	2.88 (1.60-5.75)	0.024/0.561*

NGAL, μg/mg	Day 0<br>Day 1<br>Day 3<br>Day 7<br>Day 14	56.53 (28.54-134.88)<br>-<br>-<br>61.46 (12.77-159.53)<br>307 (23.70-704.96)	2081 (630-5170)<br>1128 (454-3908)<br>1253 (136-3888)<br>1534 (229-3935)<br>993 (161-3917)	757 (261-3017)<br>783 (285-6523)<br>1551 (301-15339)<br>862 (59-7059)<br>474 (108-2854)	0.000/0.123*<br>/0.934*<br>/0.439*<br>0.000/0.959*<br>0.044/0.561*
TNF-alpha, pg/ml	Day 0	19.90 (13.00-25.60)	35.40 (29.10-52.20)	39.40 (24.50-54.70)	0.000/0.830*
IL-6, pg/ml	Day 0	68.00 (40.15-136.75)	75.20 (35.70-138.90)	86.70 (41.90-207.30)	0.444/0.311*
IL-10, pg/ml	Day 0	22.90 (15.05-33.20)	23.00 (19.60-39.10)	37.40 (26.40-64.08)	0.033/0.057*

*Comparative live and deceased.

Table S7. Univariate logistic regression analysis for mortality.

	Univariate		
Characteristic	OR	95% CI	*p*-Value
Age, per year	1.048	1.002-1.096	0.041
Male, vs female	2.143	0.622-7.387	0.227
Body mass index, per kg/m^2	0.999	0.948-1.053	0.997
Charlson index, per point	1.011	0.756-1.352	0.941
SOFA score >8, vs not	0.968	0.755-12.42	0.800
Ferritina ≥500, vs not	2.536	0.585-10.990	0.213
Use of vasopressor, vs not	1.978	0.513-7.635	0.322
Dexamethasone therapy, vs not	1.143	0.415-3.148	0.796

Table S8. Univariate and multivariate logistic regression analysis for mortality.

Characteristic	Univariate			Multivariate		
	OR	95% CI	*p*-Value	OR	95% CI	*p*-Value
Age, per year	1.048	1.002-1.096	0.041	1.411	0.329-6.050	0.643
Male, vs female	2.143	0.622-7.387	0.227	1.049	0.991-1.110	0.098
PaO_2/FiO_2 ratio, per -0.011	0.989	0.977-1.001	0.062	0.992	0.976-1.007	0.298

Abbreviations. OR, odds ratio; 95%CI, confidence interval at 95%; PaO_2/FiO_2 ratio, ratio of arterial oxygen partial pressure to fractional inspired oxygen.

Table S9. Area under the receiver-operating characteristics curve of models (clinical variables and biomarkers) for predicting mortality.

Mortality		AUC (95% CI)	*p*-Value
**Clinical variables**			
Age, per year		0.63 (0.49-0.77)	0.085
Male, vs female		0.58 (0.43-0.72)	0.322
PaO_2/FiO_2 ratio, per -0.011		0.61 (0.47-0.75)	0.141
**MODELS**			
Age + KIM-1	Day 0	0.53 (0.37-0.70)	0.703
Age + NGAL		0.62 (0.46-0.78)	0.144
Age + SerpinA3*		0.64 (0.49-0.80)	0.089
Age + SerpinA3**		0.59 (0.43-0.75)	0.271
Age + IL-6		0.60 (0.45-0.74)	0.190
Age + IL-10		0.73 (0.60-0.86)	0.002
Age + TNF-alpha		0.58 (0.43-0.73)	0.290
Age + SerpinA3*	Day 7	0.49 (0.31-0.67)	0.878

Age + SerpinA3*	Day 14	0.56 (0.35-0.77)	0.561
Male + KIM-1	Day 0	0.68 (0.53-0.84)	0.029
Male + NGAL		0.63 (0.47-0.79)	0.123
Male + SerpinA3*		0.64 (0.49-0.80)	0.089
Male + SerpinA3**		0.54 (0.38-0.71)	0.616
Male + IL-6		0.58 (0.43-0.72)	0.304
Male + IL-10		0.64 (0.50-0.78)	0.059
Male + TNF-alpha		0.52 (0.37-0.67)	0.836
Male + SerpinA3*	Day 7	0.46 (0.28-0.64)	0.665
Male + SerpinA3*	Day 14	0.53 (0.32-0.74)	0.784
PaO_2/FiO_2 ratio + KIM-1	Day 0	0.59 (0.43-0.75)	0.271
PaO_2/FiO_2 ratio + NGAL		0.63 (0.47-0.79)	0.128
PaO_2/FiO_2 ratio + SerpinA3*		0.51 (0.34-0.68)	0.910
PaO_2/FiO_2 ratio + SerpinA3**		0.55 (0.38-0.71)	0.575
PaO_2/FiO_2 ratio + IL-6		0.46 (0.31-0.60)	0.549
PaO_2/FiO_2 ratio + IL-10		0.52 (0.37-0.67)	0.801
PaO_2/FiO_2 ratio + TNF-alpha		0.61 (0.46-0.75)	0.162
PaO_2/FiO_2 ratio + SerpinA3*	Day 7	0.59 (0.41-0.77)	0.346
PaO_2/FiO_2 ratio + SerpinA3*	Day 14	0.52 (0.28-0.75)	0.891

***ELISA, **Western blot**

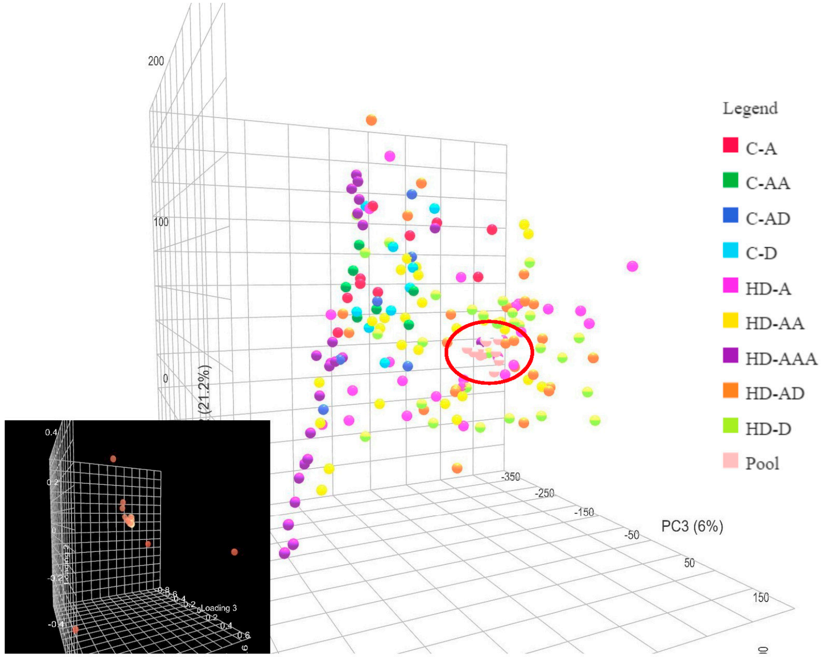

S1 Fig.

Figure S1. Principal component analysis of all the patients included in the final analysis. Red encircled spheres mark the pool quality control samples. The analysis was generated using the peak heights (sum normalized) of all the metabolites included in the metabolomic analysis.

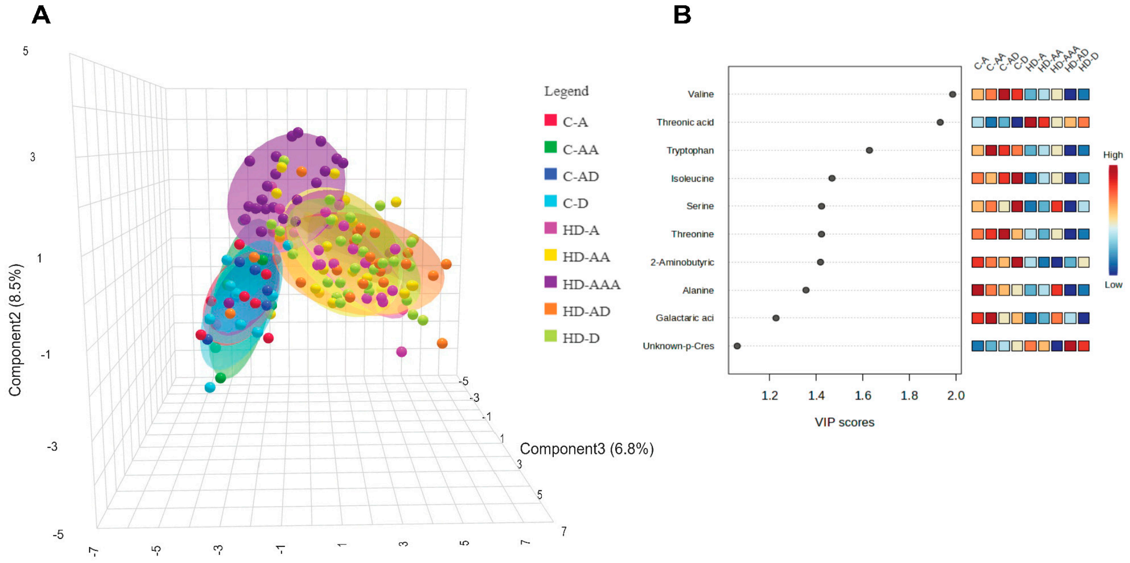

C

PLS-DA cross validation details:

Measure	1 comps	2 comps	3 comps	4 comps	5 comps
Accuracy	0.26835	0.31786	0.35479	0.35665	0.36169
R2	0.29702	0.43123	0.49766	0.5261	0.54283
Q2	0.25114	0.29931	0.29917	0.28887	0.28887

S2 Fig.

Figure S2. (**A**) PLS-DA of all the patients included in the final analysis. The analysis was generated using the peak heights after sum normalization, mean centering and log transformation. (**B**) Variable importance of projection (VIP) of the top ten metabolites. Intensity gradient is shown in the right vertical colored bar. **C**) Results of cross validation. R2: goodness of fit. Q2: predictive ability.

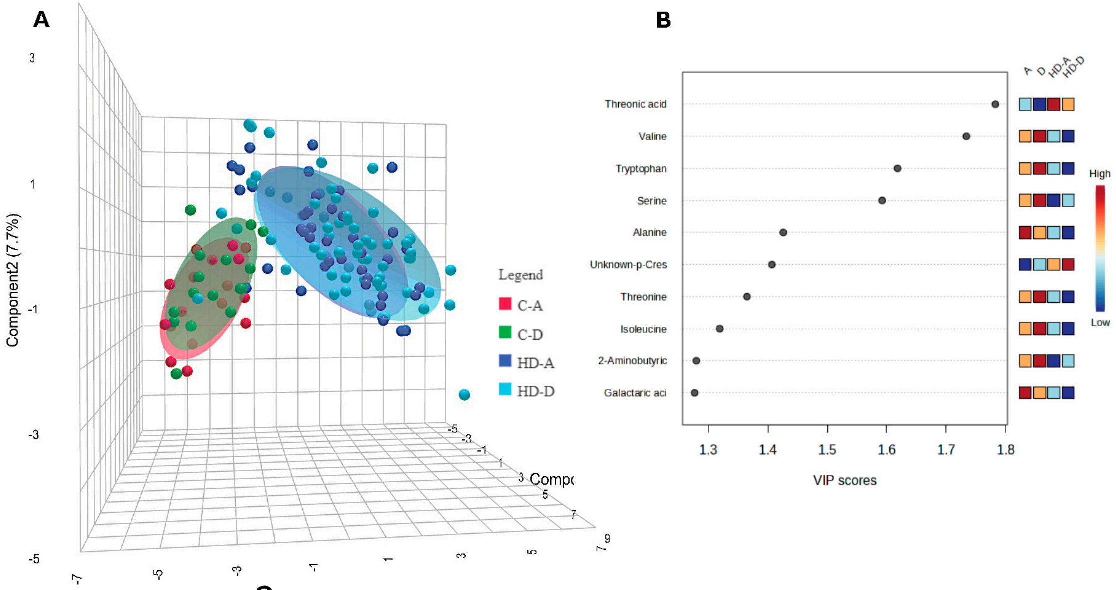

C

PLS-DA cross validation details:

Measure	1 comps	2 comps	3 comps	4 comps	5 comps
Accuracy	0.45952	0.56111	0.53148	0.56825	0.56111
R2	0.44762	0.56796	0.61544	0.64433	0.65754
Q2	0.39792	0.44393	0.47367	0.46783	0.46043

S3 Fig.

Figure S3. (**A**) PLS-DA of COVID patients considering only the KRT and fate (dead or alive). The analysis was generated using the peak heights after sum normalization, mean centering and log transformation. (**B**) Variable importance of projection (VIP) of the top ten metabolites. Intensity gradient is shown in the right vertical colored bar. (**C**) Results of cross validation. R2: goodness of fit. Q2: predictive ability.

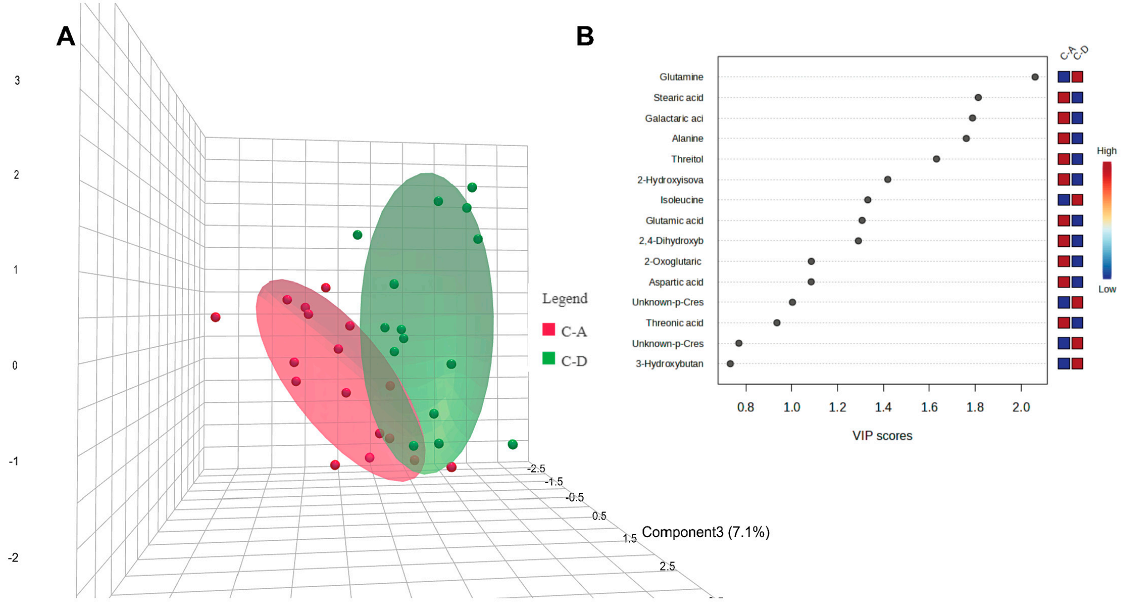

C

PLS-DA cross validation details:

Measure	1 comps	2 comps	3 comps	4 comps	5 comps
Accuracy	0.44286	0.48095	0.50476	0.4381	0.47143
R2	0.41406	0.54624	0.64753	0.70084	0.74224
Q2	-0.16348	-0.21971	-0.60052	-1.1007	-1.4872

S4 Fig.

Figure S4 (**A**) PLS-DA of COVID patients only considering the fate (dead or alive). The analysis was generated using the peak heights after sum normalization, mean centering and log transformation. (**B**) Variable importance of projection (VIP) of the top ten metabolites. Intensity gradient is represented by the right vertical colored bar. (**C**) Results of cross validation. R2: goodness of fit. Q2: predictive ability.

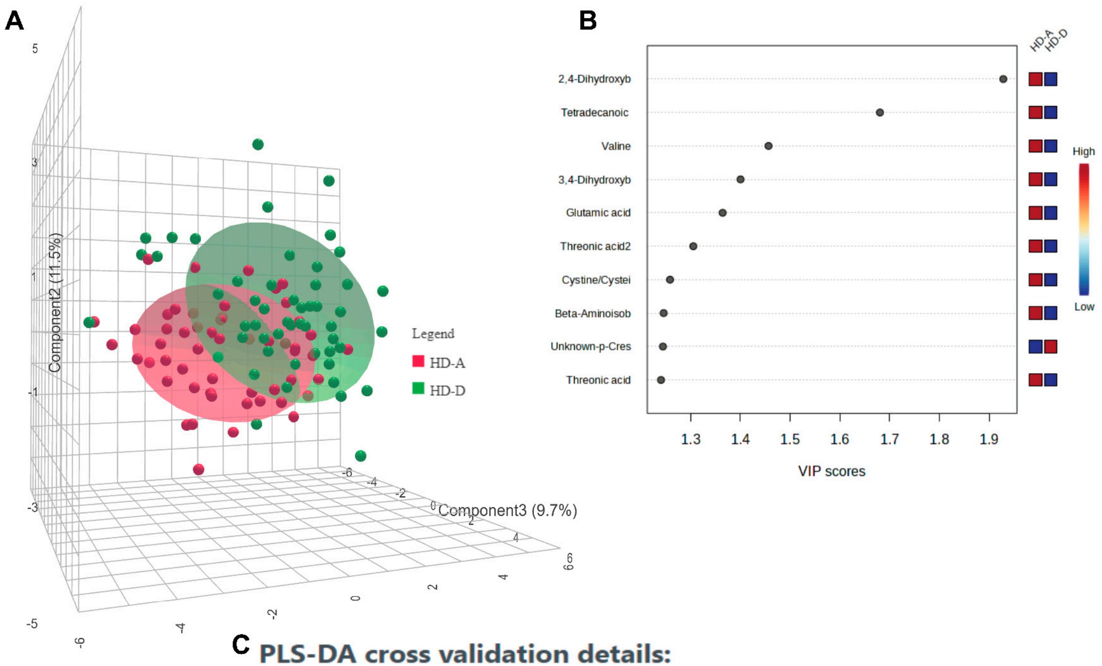

C **PLS-DA cross validation details:**

Measure	1 comps	2 comps	3 comps	4 comps	5 comps
Accuracy	0.6397	0.60065	0.62835	0.67554	0.70606
R2	0.14514	0.27706	0.36048	0.42239	0.4678
Q2	-0.0039159	-0.050659	-0.065191	-0.0525	-0.073986

S5 Fig.

Figure S5. (**A**) PLS-DA of Hemodialyzed patients considering the fate (dead or alive). The analysis was generated using the peak heights after sum normalization, mean centering and log transformation. (**B**) Variable importance of projection (VIP) of the top ten metabolites. Intensity gradient is represented by the right vertical colored bar. (**C**) Results of cross validation. R2: goodness of fit. Q2: predictive ability.
